# Food Microbial Diversity

**DOI:** 10.3390/microorganisms9122556

**Published:** 2021-12-10

**Authors:** Agapi I. Doulgeraki, Chrysoula C. Tassou

**Affiliations:** Institute of Technology of Agricultural Products, Hellenic Agricultural Organization—DIMITRA, Sofokli Venizelou 1, GR-14123 Lycovrissi, Attica, Greece

The microbiological quality and safety of food could be assessed by mapping the microorganisms present in a particular type of food. Through the years, different approaches have been adopted to monitor and characterize the microorganisms present in a certain type of food. Microbial characterization of isolated microorganisms was performed through the application of morphological and biochemical tests and/or molecular approaches [1]. In recent years, the evolution of molecular biology has led to the development of new technologies where the DNA is extracted directly from a sample and the microbiota could be considered by analyzing the massive sequences resulting from these methods [2]. The use of these advances techniques to monitor the presence, survival, behavior and characteristics of food-related microorganisms with food quality and safety aspect is reviewed [3,4,5,6,7]. Nowadays, new approaches are employed to evaluate the quality and safety of foods by collecting data from several sensors combined with data analysis using advanced mathematics [8]. The Special Issue “Food Microbial Diversity” of *Microorganisms* aimed to collect original research articles or reviews that apply culture-dependent and culture-independent technologies to study the microbial diversity of foods and/or exploit the microbial physiology and microbial properties with a view on safety and quality of foods.

In this Special Issue, one review and seven research articles are included. In these articles the microbial diversity of several foods including the plant-based foods (cherries and olives) [9,10,11], foods of animal origin (meat and dairy products) [12,13] and drinks/beverages (wine and kombucha) [14,15,16] was monitored and/or the microbial physiology and properties were exploited [11,14,16]. In these studies, the microbial characterization was performed through the application of molecular approaches to identify the isolated microbial species [12,13] and analyses of the massive sequences resulting from culture-independent approaches [9,10,11,13,15]. The mapping of microorganisms present in a particular type of food through this collection of articles revealed important information related to the quality and safety of the studied products. In brief, the microbial consortium that comprises the starter cultures used for the production of Kombucha in several brewers located in North America was explored [15]. Moreover, the technological, spoilage and pathogenic mycobiota present on cherries was characterized [11]. Similarly, Madoroba et al. [13] collected microbial quality and safety data from meat and meat products purchased from South Africa.

Three works [9,10,12] aimed to assess the validity of the hypothesis that certain taxa could serve as potential indicators of a plant cultivar, geographical origin and/or harvested-production period of foods. Indeed, the bacterial and yeast communities of three different table olives varieties harvested from certain Greek regions were characterized by metagenomic analysis [9,10]. Garroni et al. [12] characterized with genomic approach the lactic acid bacteria isolates derived from raw milk which was collected from different regions of Malta during summer and winter periods. The set of these three studies concluded that the microbial fingerprint is associated with and could be an indication of the olive’s variety [9], the geographical region of collection [9,10,12] and the period of collection/production [12]. Similarly, another study reported the effect of harvesting tree and post-harvesting processing on detected microbial taxa of cherries detected by metagenomic analysis [11].

The exploitation of microbial properties of yeasts isolated from cherries revealed that several yeast species could be used to inhibit important fungal pathogens to control the disease of the fruit [11]. In another work, the ability of different strains of an important wine spoilage yeast to form biofilm was examined and highlighted the importance of understanding the mechanism of adaptation in a winery environment [14]. Moreover, the microbial interactions that could be occurred during the wine production were reviewed [16]. These works emphasize the required knowledge related with the succession of microbial communities during fermentation to be gained for the better understanding of the fermentation process and avoiding the abnormal fermentation.

Overall, this Issue presents several aspects of food microbial diversity including the detection and characterization of technological, spoilage and pathogenic microorganisms present in food and the monitoring of starter culture succession during fermentation, microbial interactions and antagonism.

## References

[1] Doulgeraki A.I., Ercolini D., Villani F., Nychas G.-J.E. (2012). Spoilage microbiota associated to the storage of raw meat in different conditions. Int. J. Food Microbiol..

[2] Cocolin L., Ercolini D. (2015). Zooming into food-associated microbial consortia: A “cultural” evolution. Curr. Opin. Food Sci..

[3] Cocolin L., Mataragas M., Bourdichon F., Doulgeraki A., Pilet M.-F., Jagadeesan B., Rantsiou K., Phister T. (2018). Next Generation Microbiological Risk Assessment—Meta-Omics: The next need for integration. Int. J. Food Microbiol..

[4] Cocolin L., Membré J.-M., Zwietering M.H. (2018). Editorial: Integration of omics into MRA. Int. J. Food Microbiol..

[5] Den Besten H.M.W., Amézquita A., Bover-Cid S., Dagnas S., Ellouze M., Guillou S., Nychas G., O’Mahony C., Pérez-Rodriguez F., Membré J. (2018). Next Generation Microbiological Risk Assessment—potential of omics data for exposure assessment. Int. J. Food Microbiol..

[6] Haddad N., Johnson N., Kathariou S., Métris A., Phister T., Pielaat A., Tassou C., Wells-Bennik M.H.J., Zwietering M.H. (2018). Next Generation Microbiological Risk Assessment—potential of omics data for hazard characterization. Int. J. Food Microbiol..

[7] Rantsiou K., Kathariou S., Winkler A., Skandamis P., Saint-Cyr M., Rouzeau-Szynalski K., Amézquita A. (2018). Next Generation Microbiological risk assessment—opportunities of Whole Genome Sequencing (WGS) for foodborne pathogen surveillance, source tracking and hazard identification. Int. J. Food Microbiol..

[8] Tsakanikas P., Karnavas A., Panagou E.Z., Nychas G.-J.E. (2020). A machine learning workflow for raw food spectroscopic classification in a future industry. Sci. Rep..

[9] Argyri K., Doulgeraki A.I., Manthou E., Grounta A., Argyri A.A., Nychas G.-J.E., Tassou C.C. (2020). Microbial Diversity of Fermented Greek Table Olives of Halkidiki and Konservolia Varieties from Different Regions as Revealed by Metagenomic Analysis. Microorganisms.

[10] Kazou M., Tzamourani A., Panagou E.Z., Tsakalidou E. (2020). Unraveling the Microbiota of Natural Black cv. Kalamata Fermented Olives through 16S and ITS Metataxonomic Analysis. Microorganisms.

[11] Stanevičienė R., Lukša J., Strazdaitė-Žielienė Ž., Ravoitytė B., Losinska-Sičiūnienė R., Mozūraitis R., Servienė E. (2021). Mycobiota in the Carposphere of Sour and Sweet Cherries and Antagonistic Features of Potential Biocontrol Yeasts. Microorganisms.

[12] Garroni E., Doulgeraki A.I., Pavli F., Spiteri D., Valdramidis V.P. (2020). Characterization of Indigenous Lactic Acid Bacteria in Cow Milk of the Maltese Islands: A Geographical and Seasonal Assessment. Microorganisms.

[13] Madoroba E., Magwedere K., Chaora N.S., Matle I., Muchadeyi F., Mathole M.A., Pierneef R. (2021). Microbial Communities of Meat and Meat Products: An Exploratory Analysis of the Product Quality and Safety at Selected Enterprises in South Africa. Microorganisms.

[14] Dimopoulou M., Kefalloniti V., Tsakanikas P., Papanikolaou S., Nychas G.-J.E. (2021). Assessing the Biofilm Formation Capacity of the Wine Spoilage Yeast *Brettanomyces bruxellensis* through FTIR Spectroscopy. Microorganisms.

[15] Harrison K., Curtin C. (2021). Microbial Composition of SCOBY Starter Cultures Used by Commercial Kombucha Brewers in North America. Microorganisms.

[16] Zilelidou E.A., Nisiotou A. (2021). Understanding Wine through Yeast Interactions. Microorganisms.

